# Human Height Is Positively Related to Interpersonal Dominance in Dyadic Interactions

**DOI:** 10.1371/journal.pone.0117860

**Published:** 2015-02-26

**Authors:** Gert Stulp, Abraham P. Buunk, Simon Verhulst, Thomas V. Pollet

**Affiliations:** 1 Department of Psychology, University of Groningen, Groningen, The Netherlands; 2 Groningen Institute for Evolutionary Life Sciences, University of Groningen, Groningen, The Netherlands; 3 Department of Population Health, London School of Hygiene & Tropical Medicine, London, United Kingdom; 4 Faculty of Social and Behavioral Sciences, University of Curaçao, Willemstad, Curaçao; 5 Department of Social and Organizational Psychology, VU University Amsterdam, Amsterdam, The Netherlands; Indiana University, UNITED STATES

## Abstract

Across cultures, taller stature is linked to increased social status, but the potential reasons why this should be are unclear. One potential explanation is that taller individuals are more likely to win a dyadic confrontation with a competitor (i.e., they are more dominant), which leads to higher social rank. Although some previous studies have shown that perceptions of status or dominance are related to height, and are therefore consistent with such an explanation, there is surprisingly little research testing whether height actually has any influence on the behavioural outcomes in real-life social interactions. Here, we present three naturalistic observational studies demonstrating that height predicts interpersonal dominance during brief dyadic interactions. Study 1 investigated the likelihood of giving way in a narrow passage (*N = 92*); Study 2 investigated giving way in a busy shopping street, plus the likelihood of colliding with another individual (*N* = 1,108); and Study 3 investigated the likelihood of maintaining a linear path while walking, and potentially entering another individual’s personal space (*N* = 1,056). We conclude that human height is positively related to interpersonal dominance, and may well contribute to the widely observed positive association between height and social status.

## Introduction

Both historically and cross-culturally, the term “big man” has been used to denote an individual of both high social status and physical stature. According to Ellis [1, p.279], the phrase is ‘*a conflation of physical size and social rank and … “big men” are consistently big men*, *tall in stature*’ (see also [2]). For most of human evolution, it seems likely that “big men” experienced increased social status (i.e. increased access to resources) due to their physical superiority in competition with others. Although, historically, size may have been a more important determinant of male status (reflected in the average difference in height between the sexes, and the fact that historical and ethnographic sources refer exclusively to big men), the relation between height, social status and power is obviously applicable to women as well, especially in societies with greater gender equality. Indeed, among contemporary human populations, height is positively related to proxies of social status, such as leadership, professional achievement, education, and income [3–9] in both men and women.

Despite the overwhelming evidence that human stature is positively related to social status in both men and women in Western societies, the proximate mechanisms underpinning this phenomenon remain obscure. Several hypotheses to explain this relationship have been proposed, including the increased cognitive ability associated with greater height (explained by factors such as genes or nutrition: [6]), the increased health problems associated with shorter stature [10], and the observation that taller individuals appear to experience better childhood environments (i.e. parental resources; [11]). All these hypotheses, however, interpret the correlation between height and social status to be indirect; that is, this relationship is mediated by factors like improved nutrition and health, that are both a cause and consequence of higher social status in and of themselves. Interestingly, Persico and colleagues [11] show that the higher social status of taller individuals persists even after controlling for the above factors, suggesting that height could have a direct influence on the ability to achieve high social status in contemporary, industrialized society (see also [12]). Moreover, findings suggesting that taller individuals achieve greater levels of upward social mobility [13–15], even when familial circumstances are very similar (e.g., sibling pairs: [16,17]), gives further credence to the idea that the positive association between height and status may be independent of childhood circumstances.

Here, we consider the possibility that height *directly* influences the likelihood of attaining higher social status. More specifically, we hypothesize that taller people achieve higher social status as a result of their increased interpersonal dominance during confrontations with competitors. Dominance in the animal kingdom is defined as 'an attribute of the pattern of repeated, agonistic interactions between two individuals, characterized by a consistent outcome in favour of the same dyad member and a default yielding response of its opponent rather than escalation’ [18, p. 283]. Although dominance as such is a relative measure (based on repeated interactions), and not an absolute property of an individual, in this study we will refer to interpersonal dominance as the likelihood of an individual winning a dyadic confrontation. We hypothesize that the probability of winning a confrontation increases with height of the individual in relation to their opponent. The form and function of such confrontations can be as diverse as the society in which they occur, and although the advantage of winning one confrontation may be small, the cumulative effect of many such advantages may be instrumental to achieving higher social status.

The hypothesis that body size is related to dominance echoes findings in the animal kingdom. Darwin was among the first to suggest that males were larger than females in most mammals because such large size was advantageous in contests over mates [19, p. 260], and later studies have confirmed that size is indeed important in intra-sexual competition. Among mammals, larger males are usually more likely to win fights from smaller males [20], which leads to higher social rank and increased social dominance, and, consequently, increased access to females [21,22]. Recently, Puts [23] argued that, although inter-sexual selection (i.e. mate choice) has been considered the main driver of sexual selection in humans, differences in body size, strength, and aggressiveness between the sexes are probably better explained in terms of intra-sexual competition. Thus, sexual dimorphism in stature may well be a consequence of past intra-sexual competition between males.

Among humans, there is also some evidence to suggest that height is related to physical dominance [21] (although observed relationships are often weak): taller compared to shorter men are physically stronger and perceived to be stronger [24]; physically more aggressive [25]; show better fighting ability [24–26]; and feel less threatened by physically dominant men [27]. However, physical strength and fighting ability may seem unlikely determinants of social status in modern Western societies, given that individuals are prohibited by law from using force against another individual [23]. Nevertheless, we suggest that height is associated with dominance in contemporary populations, resulting in taller individuals being more likely to win (non-physical) confrontations against shorter individuals, albeit in more subtle ways.

How, then, could human height directly influence the probability of winning non-physical confrontations? First, even though the use of force is prohibited by law, the increased physical strength [24] and fighting ability [26] of taller individuals may be perceived as more threatening during a contest [24], even when that contest is non-physical. Taller people are also perceived as more competent, authoritative, intelligent, dominant, and having better leadership qualities [9,28–34]. Such height-dependent perceptions may then contribute to the increased dominance of taller individuals if shorter individuals act on their perceptions, and treat those who are taller as more competent, authoritative, and dominant than they are, and so yield to them in competitive situations.

Height may also affect how people perceive themselves, and so influence behaviour (which as noted, in part reflects how other people treat them). For instance, taller individuals, particularly taller men, have higher levels of self-esteem than shorter individuals [9] and are more likely to see themselves as leader [35], which may result in taller individuals displaying more self-confidence in social interactions. Increased self-esteem may itself be a consequence of experiencing more favourable contest outcomes earlier in life. Children as young as ten months old recognize that size plays a role in dominance contests [36], and there is some evidence to suggest that taller individuals win more contests/confrontations during childhood and young adulthood than shorter individuals: taller children win more aggressive bouts on the playground [37] and are less likely to be a victim of bullying [38]. It has also been shown that taller teenagers participate more in social activities, which in turn has been shown to have long-term effects on social status in later life [11]. Thus, the cumulative effects of the positive contest outcomes experienced by taller individuals throughout development are likely to contribute to increased self-esteem and hence increased dominance in adulthood.

Despite the clear positive association between height and social status, and the well-established perceptual links between height, dominance, and status, there are only a handful of studies that consider how height influences behavioural outcomes in social encounters. For instance, Huang and colleagues [39] showed that, during a negotiation task, individuals perceived to be taller were also more influential: when competitors were perceived to be tall (by being filmed from below), they had more influence during the task, than when competitors were perceived to be short (by being filmed from above). Similarly, individuals assigned taller avatars in a virtual reality setting behaved more selfishly in economic games than those assigned shorter avatars [40,41]. Finally, the finding that taller referees displayed greater authority during football matches, was interpreted as reflecting the increased dominance or status of these taller individuals [42].

In this paper, we extend these findings and examine whether stature is positively related to interpersonal dominance in subtle non-physical contests, via a series of observational studies. In Study 1, we examined whether height influenced the probability of yielding to another individual when passing through a narrow passageway. Imagine a situation where two individuals from opposite directions simultaneously attempt to pass through a narrow passageway that only accommodates the passing of a single individual at a given time. Which individual is more likely to take precedence and which individual is more likely to give way? We hypothesized that, in a real-life situation, this game of ‘chicken’ (e.g., [43]) would result in taller individuals being more likely to take precedence, with shorter individuals being more likely to give way, so allowing taller individuals to pass first.

In Study 2, we investigated whether people gave way to confederates of varying height, who walked against the stream of pedestrian traffic in a busy shopping street. On busy shopping streets, people walk in a variety of directions at a variety of speeds heading toward a variety of destinations. Yet, for the most part, people obey an implicit rule that they should walk on the “correct” side of the street (either right or left, depending on the country). As a result, pedestrian traffic self-organises, and the overwhelming majority of people on the same side of the street will walk in the same direction. What happens when an individual violates this norm and walks against the flow of pedestrian traffic? More pertinently to our aims here, does the height of the person violating this norm influence how people react? We therefore investigated whether pedestrians would be more likely to give way to, and less likely to bump into, a taller individual who walked against the flow of pedestrian traffic than they would to a shorter individual.

In Study 3, we examined whether the height of a pedestrian influenced his or her behaviour towards a confederate who was partially blocking the pedestrian’s pathway. In general, people try to avoid invading someone else’s personal space, and ensure they pass by others at a socially acceptable distance. What happens, however, when an unknown individual partly blocks your pathway? Do people choose to remain on their original path, thereby passing by such individuals in close proximity, or do they divert from their chosen path, thereby giving a wider berth to the blocking individual? In this study, we tested whether the height of the passing pedestrian, would significantly influence the path chosen. We hypothesized that taller pedestrians would be less likely to yield and divert from their path. Thus, in all three studies, we hypothesized that height would be positively related to dominance, such that taller individuals would be less likely to yield than those who were shorter.

## Method

All the research reported in this document was approved by the psychology ethics committee of the University of Groningen, which decided that no informed consent was needed. All studies had an observational nature, with observations conducted in public areas where any person could reasonably expect to be observed, and data gathered were evidently anonymous. All studies were performed in a mid-size city in the north of the Netherlands. The average height for men and women aged in their early 20s in this region is approximately 185.6 and 172.4 cm [44]. Although we do not have the weights of the confederates, all confederates were in the ‘normal’ BMI range. All observers were aware of the aims for each study. All analyses were performed using R [45], version 3.1.1.

### Study 1—Taking precedence and giving way on a narrow sidewalk

#### Procedure

**Fig 1:**
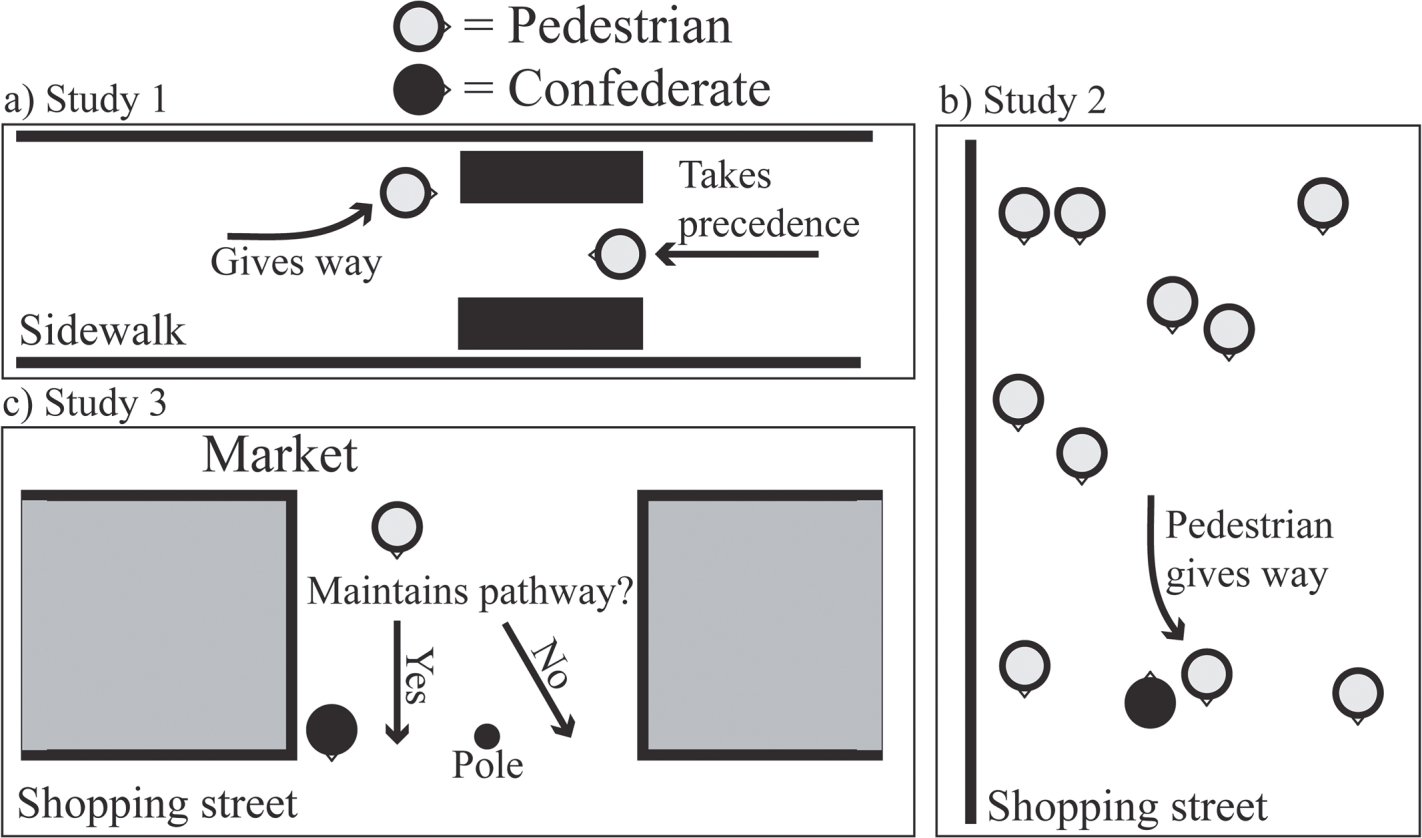
The set-up from (A) Study 1, (B) Study 2, and (C) Study 3.

We observed pedestrians entering and leaving a supermarket. To do so, pedestrians had to walk through a narrow passage on a sidewalk (Fig. 1A). The passage was too narrow for two individuals to pass through simultaneously. Thus, when two individuals approaching from opposite directions attempted to pass, one individual was required to give way (Fig. 1A). In the first part of our experiment, we made use of narrowness of passageway resulting from temporary scaffolding (because of construction work). After the scaffolding was removed, we used bicycles to create a similarly narrow passage. All observations were performed by pairs of observers (comprised of a total of six different observers). The observers stood on the opposite side of the street, outside of the direct line of sight of the pedestrians. For each pair, the observers agreed on both the height and age of each individual, and on which individual took precedence and which individual gave way. Individual height was estimated using chalk lines marked on the wall next to the passageway. The lines were marked in ten cm increments from 160 to 200 cm. A pilot experiment demonstrated that this method of estimating height was reliable, as high inter-rater reliability correlations across all raters were found (all Pearson *r* > .95; *p* < .0001). Groups and individuals pushing either bicycles or buggies were not included in the observations.

#### Analyses

In total, we observed 92 pairs of individuals trying to pass through the passageway at exactly the same time on six different observation days (during 12.00–13.30 and 17.00–19.00, mid-April 2012). We only included same-sex pairs (*N* = 50 pairs). Heights were estimated to be equal in 4 of these 50 pairs, and these were excluded from the analyses, leaving 46 pairs (28 male pairs and 18 female pairs). The perceived ages of these individuals were between 16 and 75. A paired samples *t*-test was used to test whether those who took precedence were taller than those who yielded and gave way. To test for differences in the effect of height depending on the sex of the pair, we used a General Linear Model, with the difference in height between the individuals as a dependent variable and sex as a fixed factor. This analysis is equivalent to a paired samples *t*-test when no fixed factors are included in the GLM and only an intercept is fitted. Because age is related to height and differences in age between the individuals in the pair may influence who yields, we also controlled for the difference in perceived age in the GLM. Additionally, we reran the analyses only including couples in which the perceived age differences did not exceed 15 years. Including the pair of observing experimenters as a random effect did not influence the results, nor did the method by which the passageway was narrowed (scaffolding versus bicycles; results not reported).

### Study 2—Giving way and collisions in a busy shopping street

#### Procedure

Confederates of varying height walked up and down a crowded shopping street. They were instructed to walk in a straight line, against the flow of pedestrian traffic (i.e. walking on the left side of the street) and to not look oncoming pedestrians in the eye, but to gaze either at shop windows or into the middle distance (looking around, as it were). One observer (of which there were six in total; the same individuals also acted as confederates) observed the sex of each pedestrian encountered, whether the pedestrian gave way to the confederate (i.e. the pedestrian would move to one side and onto a different heading, in order to avoid a collision with the pedestrian), and whether the pedestrians collided with the confederate (Fig. 1B). We defined a collision as any physical contact between a pedestrian and the confederate. When it was evident that the pedestrian was not going to step aside for the confederate and a collision was imminent, the confederate would then step aside and avoid contact as best as possible. When a collision occurred, the confederate would apologize to the pedestrian. In 70% of the cases in which the pedestrian did not give way, there was some form of physical contact with the confederate (such as the arms bumping slightly into each other). Even in cases were the pedestrian gave way to the confederate (by diverting from his or her path), physical contact still occurred (in 25% of the interactions). The behaviour of the confederates with respect to collisions was not easily standardized, and individual differences in behavioural dispositions may have affected the rate of collisions. Heights and ages of the pedestrians were not recorded, as this was too difficult to assess accurately by the experimenter, who also had to maneuver through the busy shopping street, and avoid colliding with pedestrians. All confederates were dressed in a similar fashion (jeans and dark jacket). Eight female confederates (with heights of: 160, 161, 171, 172, 175, 177, 183 and 183 cm) and seven male confederates (with heights of: 170, 177, 180, 185, 200, and 200 cm) participated in the study. Pedestrian couples were not included. Observations were made on eleven different days (at peak hours for pedestrian traffic; 14–17 and 19–21 on Thursday evenings).

#### Analyses

Logistic mixed models were used to analyse the data, using the lme4 package [46]. The binomial dependent variables were (a) whether the pedestrian gave way to the confederate (i.e. stepped aside) and (b) whether a collision occurred. As independent factors, we included confederate height and sex, and the sex of the pedestrian. Confederate identity was included as a random factor because observations within a confederate cannot be assumed to be independent. Including the identity of the observer as a random factor did not change our results (results not reported). We determined the (pseudo-) *R* ^*2*^ for the full model (i.e. *conditional R* ^*2*^; proportion of variation explained by both fixed and random effects) based on the methods by Nakagawa & Schielzeth [47], using the MuMIn package [48]. Furthermore, we determined the *R* ^*2*^ of the effect of height for each sex (i.e. the *marginal R* ^*2*^; proportion of variation explained by fixed effects), to compare their magnitude.

### Study 3—Maintaining one’s pathway in a narrow passage

#### Procedure

The study was set in a passageway for pedestrians between a market and the main shopping street of the city. The passageway was narrow (approximately 2 m wide) and contained a small pole in the middle of the passage near the shopping street (Fig. 1C). The pole acts as a ‘guide’ to ensure people walk through the passage on the ‘right’ side. Thus, people coming from the market and entering the shopping street mostly walk on one side of the passage (and pole), whereas people going to the market from the shopping street usually walk on the other side of the passage (and pole; Fig. 1C). Taking advantage of this set-up, we positioned a confederate in a way that partially blocked the passage for those pedestrians walking from the market towards the shopping street. More specifically, the confederate was asked to lean against the wall in the vicinity of the pole, thus leaving only around one meter of space between the confederate and the pole through which pedestrians could pass. We examined whether pedestrians would maintain their original path, and so pass the confederate at sufficiently close proximity to invade their personal space (Fig. 1C), or whether they would yield to the confederate by deviating from their original path (and so passing the confederate on the ‘wrong’ side of the pole). This set-up thus provided a clear and unambiguous measure of path deviation by allowing us to record simply on which side of the pole a given pedestrian chose to walk in order to pass through the passage. Observations were conducted on ten different days (between April 24^th^ and June 5^th^ 2012; between 11.00–17.00 h).

In each observation session, the blocking confederate was instructed to lean against the wall, with his or her right arm resting against the wall, so that they were facing towards the shopping street and away from the pedestrian. They were instructed to play with a mobile phone to make their behavior appear more ‘natural’. Four female confederates (with heights of 171, 175, 176, and 183 cm) and three male confederates (with heights of 177, 185, and 200 cm) participated in the study. As the main focus of the study was the height of the pedestrians, rather than that of the confederates (as was the case in Study 2), we used fewer confederates, and their individual heights did not cover the entire height range. It is possible, however, that confederate height may influence the behavior of the pedestrians, and therefore we included it in our analyses.

Two observers simultaneously recorded the behavior of the pedestrians coming from the market and walking through the passage, approaching the confederate from behind. One researcher recorded the height, sex and perceived age of each pedestrian, whereas the other researcher recorded whether or not pedestrians maintained their path (i.e. they recorded which side of the pole the pedestrian chose to pass the blocking confederate). The observers were positioned behind a corner, out of the line of sight of the pedestrians. To our knowledge, pedestrians were completely unaware of the presence of the observers while walking through the passageway. Individuals walking in groups or with a bicycle or a buggy were not recorded. We also did not record the behaviour of pedestrians when other pedestrians were walking through the passageway, as this resulted in further blocking of the pathway in addition to our confederates, and the basis of pedestrian movement decisions with respect to the confederate became ambiguous. In total, 1,056 pedestrians were observed passing by our confederates.

Due to local conditions of this experimental-set up, we could not make use of chalk markings on the wall to estimate pedestrian height. Instead, observers estimated height without any reference points. Although this method is less accurate than the one in our first study, we do not consider this to be a major problem, for two reasons. First, all our research assistants were trained during our first study to make accurate height estimations. Second, two researchers rated a subset of pedestrians on height, and inter-rater correlation was high (Pearson *r* = .83, *p* < .0001, *N* = 50). The perceived ages of the pedestrians were between 11 and 80.

#### Analyses

We used logistic mixed models to analyse the data, with the chosen path of the pedestrian (i.e. whether the pedestrian was observed to deviate from his or her path) as the dependent variable. We included height and sex of the pedestrian, and the sex of the confederate, as fixed effects, and we included confederate identity as a random effect because observations within a confederate may not be independent. Including observer identity as a random effect did not change our results (results not reported). We standardized the estimated height of pedestrians within each sex in order to better compare the effect of height between the sexes: a shift of one standard deviation therefore means the same for both men and women in this study. For more details pertaining to the analyses, see the ‘Analyses’ section of Study 2.

## Results

### Study 1—Taking precedence and giving way on a narrow sidewalk

**Fig 2:**
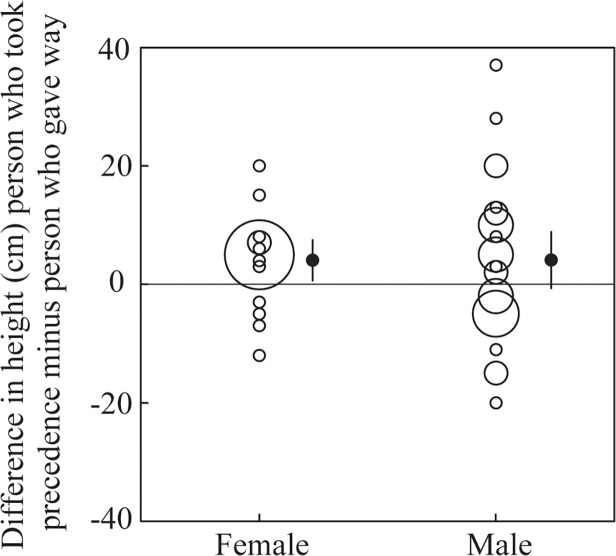
Results of Study 1. Priority of access in relation to difference in height (cm) (individual who took precedence—individual who gave way) for female and male pairs. The diameter of the open circles indicates sample size. The black dots and bar represent the mean and 95% confidence interval.

Men who took precedence were estimated to be 181.32 (*SD* = 10.77) cm in height, on average, whereas men who gave way were estimated to be 177.21 (*SD* = 5.55) cm. Similarly, women who took precedence were estimated to 171.11 (*SD* = 7.59) cm tall on average, whereas women who gave way were estimated to be 167.06 (*SD* = 6.23) cm. Combining male and female pairs revealed that individuals who took precedence were significantly taller (4.09 (*SD* = 10.96) cm) than those who gave way (paired samples t-test: *t*(45) = 2.53; *p* = .015; *d* = 0.37; Fig. 2). Similarly, taller individuals (67%) were significantly more likely than shorter individuals (33%) to take precedence (Binomial test: *N* = 46; *p* = .026).

A GLM with the difference in height as a dependent variable, revealed that there was neither a significant effect of sex (*F*(1, 44) < .001; *p* = .99; *partial η* ^*2*^ < .01), nor could this effect be attributed to the difference in perceived age (*F*(1, 44) = .65; *p* = .42; *partial η* ^*2*^ = .01). In other words, the strength of the effect of height was similar for men and women and was not driven by the effect of age. Restricting the analyses to pairs where the perceived age difference was estimated to be less than 15 years resulted in a stronger effect of height (mean difference = 5.66 cm (*SD* = 10.74); *t*(31) = 2.98; *p* = .006; *d* = 0.53). Again, there was no significant sex difference with respect to height (*F*(1, 30) < 1.900; *p* = .18; *partial η* ^*2*^ = .060), although the effect of height was, on average, 5.25 (*SE* = 3.809) cm stronger for men. Similarly, with this age range restriction, taller individuals were even more likely (75%) than shorter individuals (25%) to take precedence (Binomial test; *N* = 32; *p* = .007).

### Study 2—Giving way and collisions in a busy shopping street

**Fig 3:**
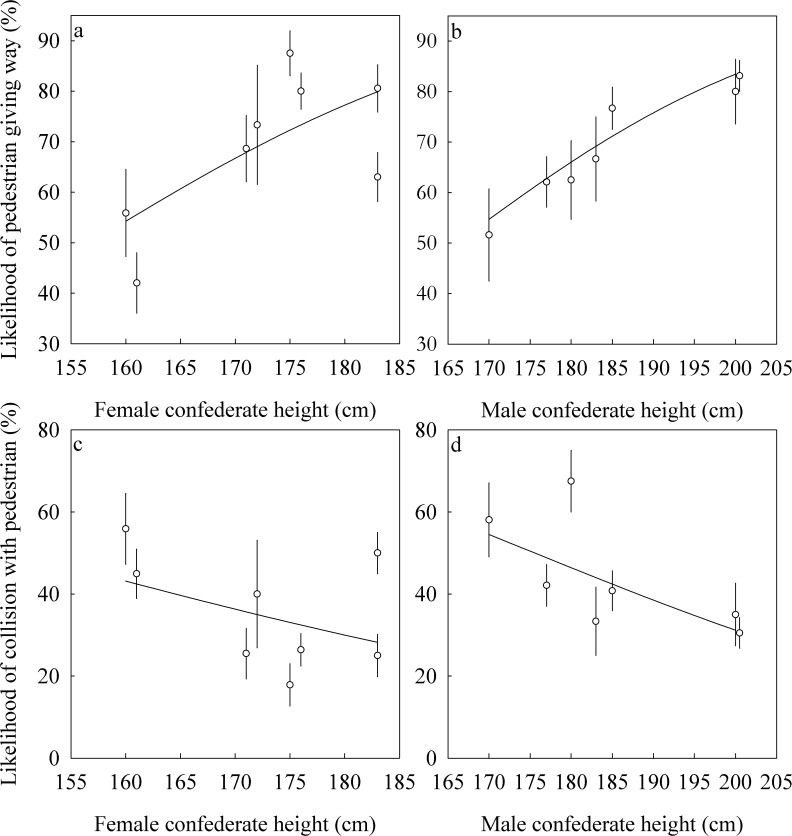
Results from Study 2. The effect of confederate height on the likelihood that a pedestrian gave way (top panels; A, B) or collided with (bottom panels; C, D) a female confederate (left panels; A, C) or male confederate (right panels; B, D).

In total, we observed 1,018 pedestrians in the shopping street. Controlling for height, we found that pedestrians were more likely to give way to female than to male confederates (Table 1). For a woman of 180 cm, 76% of individuals were predicted to step aside, whereas for a man of the same height, the value was 65%. Height was positively related to the likelihood of giving way by the pedestrian in both sexes (Fig. 3A, 3B). For our shortest female (160 cm) and male (170 cm) confederates, our model predicted that 55% and 54% of pedestrians would step aside, respectively. In contrast, for our tallest female (183 cm) and male (200 cm) confederates, this value was increased to 79% and 84% respectively. No significant interaction was found between confederate height and sex (*p* = .87). Examining the amount of variation explained only by height, we found that 7.0% of the variation in giving way in men was explained by height, whereas for women this value was 4.8%. The sex of the pedestrian had no effect on the chance of giving way (*p* = .97), nor did it interact with either confederate height (*p* = .18) or the sex of the confederate (*p* = .38). In conclusion, pedestrians were more likely to yield and give way to taller compared to shorter individuals, and this was equally true for men and women, although the effect was slightly stronger for men.

**Table 1:** Results from Study 2.

	Likelihood that confederate was given way		Likelihood of collision with confederate	
	Parameter estimate (± *SE*)	*p* value	Parameter estimate (± *SE*)	*p* value
Intercept	-7.72 ± 2.05	.0002	4.91 ± 2.28	.031
Sex confederate^a^	-0.54 ± 0.26	.041	0.56 ± 0.31	.075
Sex pedestrian^a^	—^b^	—	-0.46 ± 0.20	.022
Sex conf. x Sex ped.	—^b^	—	0.50 ± 0.28	.070
Height	0.049 ± 0.012	<. 0001	-0.031 ± 0.013	.018
Random intercept^c^	0.093 ± 0.31		0.14 ± 0.38	
Marginal *R* ^*2*^ ^d^	.059		.038	
Conditional *R* ^*2*^ ^d^	.085		.078	

Logistic mixed model parameter estimates (± *SE*) for the effect of the height, sex of the confederate, sex of the pedestrian, and their interactions on the likelihood that the pedestrian would (i) give way to the confederate or (ii) collide with the confederate (*N* = 1,108). Non-independence due to confederate ID was modelled as a random intercept.

^a^ Reference category is female

^b^ Non-significant (both *p* > .38) and therefore not included in the final model

^c^ Intercept at the level of confederate; variance estimate ± *SD*

^d^ (Pseudo-)R^2^; see text for explanation.

Confederate height was negatively related to the likelihood of a collision (Table 1; Fig. 3C, 3D). That is, pedestrians were more likely to collide with shorter confederates than with taller confederates. The lack of a significant interaction between confederate sex and height of the confederate (*p* = .81), again suggests that the effect of height was similar for men and women. Examining height only, we again found that it was more predictive in men: in men it explained 3.3% of the variation in collision probability, whereas female confederate height explained 1.5% of the variation. We also found a marginally significant interaction between the sex of the confederate and the sex of the pedestrian, such that male pedestrians were less likely to collide with female confederates (Table 1).

For our shortest female confederate (160 cm), our model predicted that 48% of women and 37% men would collide with the confederate, respectively while for our tallest female confederate (183 cm) our model predicted that only 31% of women and 22% of men would collide. There was no difference in rate of collision between the sexes when a male confederate was walking against the stream of people. For our shortest male confederate (170 cm), our model predicted that 54% of women and 55% of men respectively would collide with the confederate, whereas for our tallest male confederate (200 cm), our model predicted that only 32% of women and 33% of men would collide. There was no significant interaction between the height of the confederate and the sex of the pedestrian (*p* = .33), nor did we find a three-way interaction between the height of the confederate, the sex of the confederate and the sex of the pedestrian on the likelihood of a collision (*p* = .98). In summary, shorter confederates were more likely to collide with pedestrians than were taller individuals. In addition, male pedestrians were less likely to collide with female confederates than they were with male confederates.

### Study 3—Maintaining one’s pathway in a narrow passage

**Fig 4:**
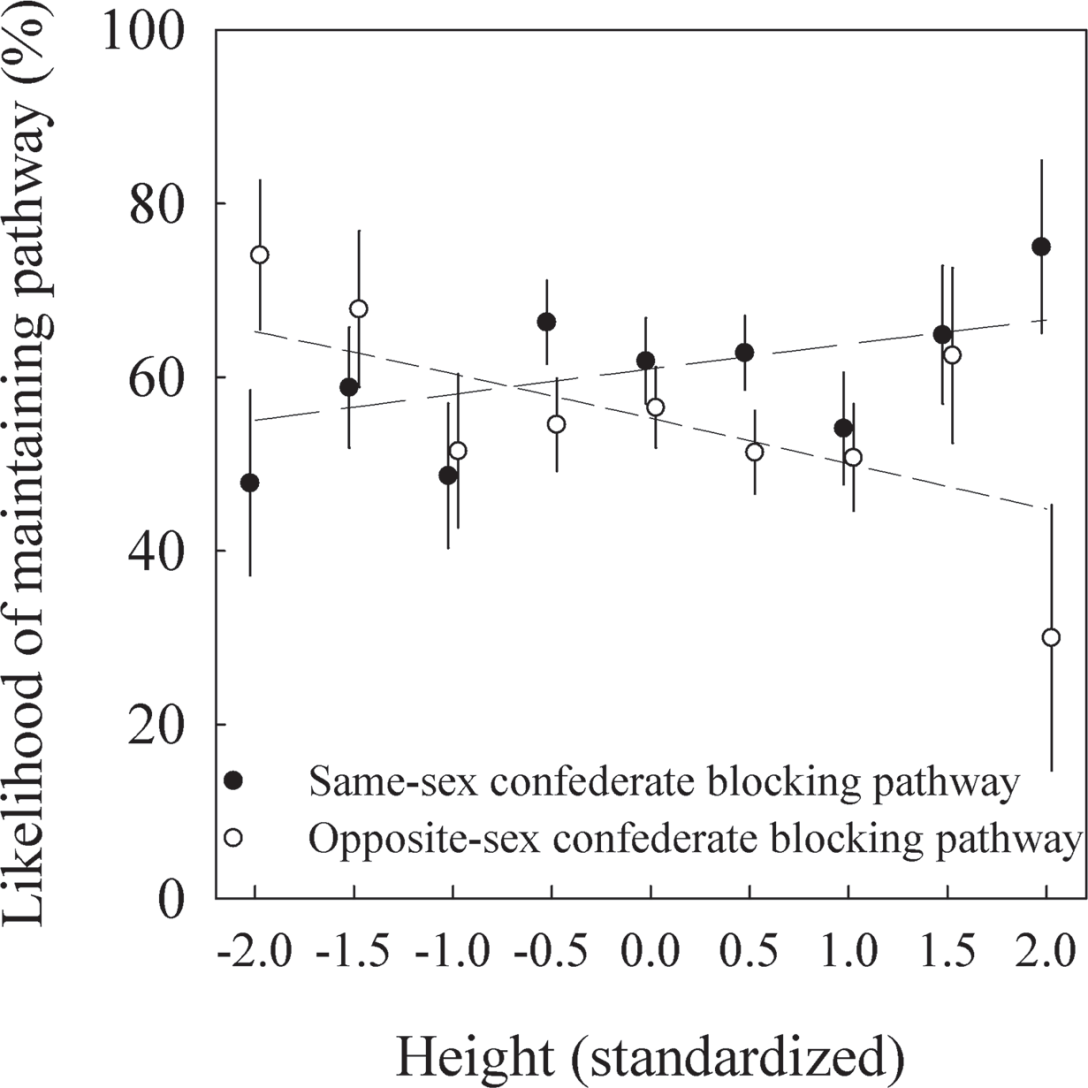
Results from Study 3. The effect of the pedestrian height (standardized) on the likelihood of maintaining one’s path (mean ± *SE*) and thereby passing close by an opposite-sex or same-sex confederate who was partially blocking the pedestrian’s pathway (see Fig. 1C).

Preliminary analysis indicated that people of both sexes behaved differently depending on whether there was a same-sex or opposite sex confederate. Rather than including the sex of the confederate in our analyses, we instead included a binary variable that specified whether the confederate was of the same sex as the pedestrian. We found a significant interaction between height of the pedestrian and confederate sex on the likelihood of passing by the confederate without deviating from their path (Table 2). When the confederate was of the opposite sex, taller individuals were more likely to yield and deviate from their path than shorter individuals (*p* = .030; Table 2; Fig. 4). A short woman (two *SD* below height) was predicted to pass by the confederate without deviating from her path with a likelihood of 68%, whereas for a tall woman (two *SD* above height) this was reduced to 49%. For men, these same values were 62% versus 41%. In contrast, when the confederate was of the same sex, there was no significant effect of height (parameter estimate for slope (± *SE*) = .12 (± .09); *p* = .17; obtained by reverse coding the variable in the analysis), although the direction of the effect was in line with our hypothesis, with taller individuals being less likely to give way. The likelihood that a tall woman would pass the same-sex confederate in close proximity without any deviation was 69% versus 56% for a short woman. For men, these values were 62% versus 51% respectively. The positive and negative slopes for pedestrian height depending on whether the confederate was of the same sex did not differ statistically in magnitude as evidenced by the overlapping standard errors of both estimates.

**Table 2:** Results from Study 3.

	Likelihood that pedestrian passed by without deviating from path	
	Parameter estimate (± *SE*)	*p* value
Intercept	0.36 ± 0.11	.002
Sex pedestrian^a^	-0.30 ± .13	.019
Confederate same-sex^b^	0.20 ± 0.13	.120
Height pedestrian	-0.21 ± 0.095	.030
Height x Confederate same sex	0.32 ± 0.13	.012
Random intercept^c^	0.005 ± 0.072	
Marginal *R* ^*2*^ ^d^	.019	
Conditional *R* ^*2*^ ^d^	.021	

Logistic mixed model parameter estimates (± *SE*) for the likelihood of passing by the confederate without deviating from path in relation to sex and height of the pedestrian, whether the confederate was of the same sex as the pedestrian, and their interaction (*N* = 1,056). Non-independence due to confederate ID was modelled as a random intercept.

^a^ Reference category is female

^b^ Reference category is ‘confederate of different sex as pedestrian’

^c^ Intercept at the level of confederate; variance estimate ± *SD*

^d^ (Pseudo-)*R* ^*2*^; see text for explanation.

This two-way interaction did not differ by pedestrian sex as evidenced by the fact that there was no significant three-way interaction between sex of the pedestrian, whether the confederate was of the same sex, and height (*p* = .47). Thus, the effect of pedestrian height on the likelihood of path deviation did not differ for male and female pedestrians. The two-way interaction between pedestrian height and same-sex confederate, however, explained twice as much of the variation in path deviation for men (*R* ^2^ = .016) compared to women (*R* ^2^ = .008). In general, men were significantly more likely to deviate from their path than women (Table 2). The perceived age of the pedestrian had no significant effect (*p* = .18). Against our expectation, the height of the confederate had no significant effect on whether the pedestrian would maintain his or her path (*p* = .72), although this model did not converge. Therefore, to better assess the effect of the relative height of the pedestrian compared to the confederate, we also ran models in which we included the difference in height between the pedestrian and the confederate for those encounters where the pedestrian was blocked by a same-sex confederate, in which we included the sex of the pedestrian in order to assess whether there was any difference in response in male versus female dyads. Relative height did not have a significant influence on the likelihood of path deviation (Parameter estimate (±SE) = 0.014 (±0.009); *p* = 0.11). Including a categorical variable that coded whether the pedestrian was taller (or of equal height) versus shorter than the confederate produced similar results (0.22 (±0.19); *p* = 0.26).

Overall then, for both male and female pedestrians, height was related to the likelihood of path deviation, but the effect of height was dependent on the sex of the confederate blocking the pathway. Taller pedestrians were less likely to maintain their path when the confederate was of the opposite sex compared to shorter pedestrian. No effect of height was observed when the confederate was of the same sex.

## Discussion

Our results show that height is related to interpersonal dominance in a variety of social settings, which we assessed in a series of observational studies. In our first study, we showed that taller individuals were more likely to take precedence when entering a narrow passage wide enough for only a single individual to pass. This effect was independent of both sex and perceived age. To the best of our knowledge, this is the first evidence that height differences affect the outcome of a brief dyadic interaction in a naturalistic setting. Given the nature of the observational set-up, we were, however, unable to assess whether this effect was because taller individuals actively take precedence, shorter individuals are more likely to give way, or both.

In a follow-up study, therefore, we investigated how pedestrians reacted towards confederates of varying height, as they walked along a busy shopping street. Pedestrians were more likely to yield to taller than to shorter confederates by giving way and stepping aside. This was equally true for both male and female confederates. In addition, when examining a more confrontational measure of dominance—actual physical contact—we found that taller confederates were less likely to collide with pedestrians than shorter ones. In line with the findings of Study 1, therefore, we found that an individual’s height influenced strongly the behaviour of others in a dyadic encounter in a naturalistic setting.

In our third study, we assessed yet another behavioural measure of dominance: the social distance adopted by people of different heights when passing by an unknown individual in a confined space. We hypothesized that when pedestrians were confronted by an individual of the same sex partially blocking their pathway, taller individuals would be less likely to yield and so more likely to pass by within closer proximity than shorter individuals. Although our findings were suggestive of this, the effect was not significant for confrontations between same sex individuals. Moreover, we found exactly the opposite pattern to that predicted when we looked at cases where an opposite-sex individual was blocking the pathway: taller pedestrians were more likely to deviate from their path than were shorter individuals. The finding that pedestrians react differently to confederates depending on their sex (also apparent in Study 2) is not surprising. It seems entirely reasonable to expect that, in same-sex interactions, competition will be more pronounced, whereas gender norms and mate choice concerns are more likely to dominate in opposite-sex interactions. As an example of such a norm, we observed in Study 2 that male pedestrians were less likely to collide with female than male confederates. Similarly, previous studies have shown that interpersonal attraction are related to proximity between two individuals [49,50], such that those attracted to one another are in closer proximity.

One potential explanation for why height should be related to individual behaviour in opposite-sex encounters relates to the absolute increase in physical size of taller men and women, not only in the vertical dimension, but also in the horizontal dimension (due to allometry). Taller and, all else being equal, wider individuals (see e.g., [51]) perhaps choose to pass by the confederate at larger distances so as to ensure a lack of physical contact and maintain a certain minimum distance. Such ideas fit well with research [51] showing that 1) taller men have wider shoulders than shorter men (indeed, the authors believed there to be a somewhat ‘universal’ ratio between height and shoulder width); 2) taller men required larger shoulder movements to move through small apertures; and 3) judgments of ‘passable’ apertures relied on eye height (and thus height). Thus for our study it may be that, because taller men and women perceive that they are more likely to pass the confederate at an unacceptable (or at least uncomfortable) degree of proximity, they instead choose to deviate from their original pathway in order to ensure that this does not occur. In contrast, shorter individuals, who are also less likely to be wide, may be able to pass by the confederate at a distance that is neither perceptually nor absolutely socially unacceptable. Although this argument is speculative, our study does provide some evidence in support: on average, men were more likely to yield and deviate from their pathway than were women. Because men are on average larger than women, the distance at which they pass by a stranger may be correspondingly higher. Indeed, our finding that men were more likely to avoid close proximity conforms to a plethora of research indicating that men require a larger amount of personal space, and greatly dislike any intrusion into this space [52,53].

We did not find a statistically significant effect of the height of the confederate blocking the passageway on the likelihood of the pedestrian to maintain its path in Study 3. One reason for this could be due to our experimental set-up, which perhaps did not tap into aspects of dominance as we assumed. In contrast to Study 1 and 2, there was no face-to-face interaction in Study 3, because all the pedestrians approached our confederate from behind. As our confederates acted ‘naturally’, by leaning against the wall, with their heads slightly tilted to look at their phone, pedestrians may not have perceived this event as a social encounter and, as such, may not have felt either dominant or submissive. Furthermore, we may have used too few confederates (e.g. three males and four females), with too little variation in height, to be able to detect statistical effects for variations in height in relation to this posture.

In conclusion, in two observational studies, we found clear evidence to support the notion that human height is positively related to interpersonal dominance (at least when that person is confronted by a same-sex individual), whereas the results from our third study were more equivocal, although we nevertheless confirm that height affects every-day behaviour.

The increased dominance of taller men and women is likely to result from both perceptions of the individuals themselves and the perceptions of others. Indeed, taller people are perceived as more dominant [9,28–30,33], and some of these biases are already apparent in very young children [36]. Perhaps because of these perceptions, pedestrians were more likely give way and less likely to collide with taller confederates compared to shorter confederates (Study 2). These different perceptions of and behaviours towards taller compared to shorter individuals may subsequently lead to increased self-esteem in taller individuals [9], which in turn is likely to affect their dominance. Indeed, an individual’s height also determined his or her behaviour towards a confederate blocking their path (Study 3). Future studies could therefore address the extent to which the relationship between height and interpersonal dominance is mediated by an individual’s direct perception of their own dominance in relation to height, versus the behaviour of others toward them in relation to their height. Manipulating height in a behavioural study with actual people (e.g., such as wearing higher shoes), without changing any other variables is difficult. Studies using virtual reality techniques may be best suited to this purpose, as the heights of individuals’ avatars can be manipulated without participants’ awareness. Some studies have already pursued this, demonstrating that, within a virtual reality setting, taller individuals made more unfair offers during economic games [40] with the behavioural effect of being virtually tall extending to negotiating more aggressively in subsequent face-to-face interactions [41].

Although the effect of height on dominance did not significantly differ between the sexes in any of our studies, the effects of height were consistently stronger for men than for women. This is in line with findings on the relationship between height and social status. While both male and female height are positively related to measures of social status [9], the magnitude of this relationship is significantly stronger for men than for women. Similarly, a recent study showed that perceptions of leadership were more closely related to height for men, than for women [32]. In addition, this study found that male height was positively associated with perceived dominance, health, and intelligence, whereas female height was associated only with perceived intelligence [32]. Height also has a differential effect on attractiveness for men and women: whereas taller men are considered more attractive, women of average height are rated as most attractive in preference studies [54,55]. Overall, then, it seems clear that taller individuals are more likely to be dominant, but male height makes a more significant contribution to this assessment than does female height, and this potentially can be explained by the relationship between height and perceptions of dominance, intelligence, health, and attractiveness [32].

A limitation of our behavioural studies is that we were only able to estimate the heights and ages of the pedestrians, rather than recording their actual heights and ages. Although perceptions of age have been shown to be highly accurate [56] and were not of central interest to our study, perceptual distortions of height in relation to status and dominance are well documented (reviewed in [33]). For instance, individuals who are higher in status, or who behave in a more dominant, risky, or aggressive fashion are perceived as taller than individuals who are lower in status or who behave submissively [29,33,57–60]. Similarly, taller individuals are perceived as more dominant than shorter individuals [29,32]. These findings may pose a problem for our observational studies, as height estimations were made during overt dominance interactions, and estimations of dominant behaviour (e.g. refusing to yield, collisions) were made while the height of the individuals involved was known (Study 1, 2). Our results could therefore be a consequence of perceptual distortions on the part of the observers, rather than an actual behavioural effect related to height. However, we believe that our results are unlikely to be a consequence of these perceptual distortions for several reasons. First, several of our measures could be easily and unambiguously assessed, such as the heights of the pedestrians relative to markings on a wall (Study 1); whether any physical contact occurred between the confederate and the pedestrian (Study 2) and which side of a pole a pedestrian would pass (Study 3). Second, it is difficult to see how perceptual distortions of height could lead to the observed interaction in our third study, as our behavioural measure of dominance was differentially affected by height, in a manner that was also dependent on the sex of the confederate blocking the pathway. For these reasons, we believe it is unlikely that our results are merely a consequence of a perceptual distortion of height in relation to dominance, or perceptual distortions of dominance on the basis of height. The use of video cameras to record interactions that can then be scored by observers blind to the aims of the study may circumvent some of these problems. It is, however, increasingly difficult to perform such studies without the awareness of the participants and ethical concerns with respect to privacy laws.

A second limitation of our behavioural studies is that all experimenters and confederates were aware of the aims of the study. It would be very difficult to devise our studies in such a way that experimenters could remain blind to these aims (particularly in Study 1 and 3). In addition, the recording of the heights (and age) of pedestrians and their behavioural interactions was taxing for observers, and adding ‘foil’ variables could compromise study accuracy and precision with respect to the key variables of interest. The aim of our three observational studies was therefore guessed easily, and we chose, therefore, to inform all experimenters and confederates. The use of video cameras may again circumvent some of these problems.

Overall, our findings suggest that, even in the absence of overt physical aggression, height influences the outcome of non-verbal confrontations between individuals. Thus, the increased social status and upward social mobility of taller individuals in modern society, usually attributed to variables such as improved health and nutrition, may occur, at least in part, as a consequence of their increased interpersonal dominance.

## Supporting Information

S1 Dataset Data from all three studies. (XLS)
